# Machine-Learning-Based Activity Tracking for Individual Pig Monitoring in Experimental Facilities for Improved Animal Welfare in Research

**DOI:** 10.3390/s25030785

**Published:** 2025-01-28

**Authors:** Frederik Deutch, Marc Gjern Weiss, Stefan Rahr Wagner, Lars Schmidt Hansen, Frederik Larsen, Constanca Figueiredo, Cyril Moers, Anna Krarup Keller

**Affiliations:** 1 Department of Clinical Medicine, Aarhus University, 8000 Aarhus, Denmark; 2 Department of Urology, Aarhus University Hospital, 8000 Aarhus, Denmark; 3 Department of Electro- and Computer technology, Section for Biomedical Engineering, Aarhus University, 8000 Aarhus, Denmark; 4 Institute of Transfusion Medicine and Transplant Engineering, Hannover Medical School, 30625 Hannover, Germany; 5 Department of Surgery, University Medical Center Groningen, 9713 GZ Groningen, The Netherlands

**Keywords:** machine learning, monitoring, pig behavior, activity tracking, animal welfare, animal experiments

## Abstract

In experimental research, animal welfare should always be of the highest priority. Currently, physical in-person observations are the standard. This is time-consuming, and results are subjective. Video-based machine learning models for monitoring experimental pigs provide a continuous and objective observation method for animal misthriving detection. The aim of this study was to develop and validate a pig tracking technology, using video-based data in a machine learning model to analyze the posture and activity level of experimental pigs living in single-pig pens. A research prototype was created using a microcomputer and a ceiling-mounted camera for live recording based on the obtained images from the experimental facility, and a combined model was created based on the Ultralytics YOLOv8n for object detection trained on the obtained images. As a second step, the Lucas–Kanade sparse optical flow technique for movement detection was applied. The resulting model successfully classified whether individual pigs were lying, standing, or walking. The validation test showed an accuracy of 90.66%, precision of 90.91%, recall of 90.66%, and correlation coefficient of 84.53% compared with observed ground truth. In conclusion, the model demonstrates how machine learning can be used to monitor experimental animals to potentially improve animal welfare.

## 1. Introduction

Animal welfare in research should always be considered due to ethical, financial, and academic reasons [1,2]. The current standard for observing pigs in experimental research is through in-person observations performed by trained animal caretakers, scoring the pig on a list of parameters [3]. The observations are standardized but could still be influenced by the subjectivity and experience of the animal caretaker [3,4]. In addition, this only allows observation of the pigs during working hours, when the staff is present, and researchers and veterinarians only have limited access to the observations in real time. Also, in-person observation methods are time-consuming, which limits the possibility of prioritizing care for sicker pigs. Considering these conditions, the practice of animal handling in experimental survival studies could arguably be improved using modern sensor technologies to quickly identify and alert staff in case of misthriving, which allows for faster and more effective treatment of the animals [4]. Eventually, this will lead to less discomfort and a higher level of animal welfare. Strategies for the implication of newer approaches to heighten animal welfare are described in the three R’s: replacement, reduction, and refinement—principles for minimizing and optimizing the use of animals in experimental research [5]. An automatic, continuous monitoring system could potentially improve the refinement principle that defines how current experimental practice could be changed to lower the number and intensity of discomforting events or how to improve the framework and experimental setup around the animals to improve the animals’ quality of life.

Recent studies on pig welfare and behavior monitoring have employed various technologies, but their focus and methodologies vary widely. Recent studies on surveillance and welfare of pigs primarily focus on pigs in the food industry with pigs living in groups in multiple pig pens, typically around seven to fifteen pigs per pen [6,7,8,9]. The pigs are surveilled not only in the stables, but also during transport and in the slaughterhouse [10]. For instance, Shuqin Tu et al. (2024) [11] used YOLOv5 to track individual pig behavior based on video data. Their approach achieved high accuracy in detecting individual pigs in complex environments, yet their system focuses on group-housed pigs, and while they used real-time video for tracking, their solution is computationally intensive and requires significant processing power, which may not be feasible in low-cost settings. Similarly, Bhujel et al. (2021) [12] applied deep learning techniques to monitor pigs’ physical and temporal activities under varying environmental conditions, demonstrating robust performance with automatic activity classification. However, their system also focuses on pigs in group housing and does not emphasize real-time, in situ monitoring or edge computing, relying on existing video datasets and higher-end computational platforms, which limits its application to more resource-constrained environments.

Larsen et al. (2021) [10] discussed various information technologies for welfare monitoring in pigs, focusing on the integration of sensors to monitor behavior. While their study considers a broader range of sensor technologies, it does not provide detailed real-time monitoring solutions for individual pigs in single pens, nor does it explore low-cost, edge device implementations for local alerting systems. Additionally, Zhang et al. (2019) [6] developed an automatic pig detection and tracking system for group-housed pigs using video data, achieving successful individual pig tracking with an accuracy of 90%. However, their system was designed for multi-pig pens and does not address the specific needs of single-pig pen environments or the use of low-cost edge devices.

Moreover, the study by Yin et al. (2025) [13] combined statistical analysis with machine learning models to classify pig health status based on hourly activity data, which was achieved with promising accuracy levels, but the system focused on group-housed pigs and did not incorporate real-time, in situ video monitoring. Likewise, Zhang et al. (2021) [14] explored activity recognition in pigs in relation to environmental conditions like greenhouse gas concentrations, using deep learning models for activity classification. While their system offers promising detection accuracy, it does not address the real-time monitoring of pigs in single pens or the use of edge devices, limiting its practicality in low-cost, real-time applications.

In contrast, our contribution centers on real-time, video-based monitoring of pigs in single-pig pens, specifically using a low-cost edge device with a local alerting micro-service architecture that is required to be independent of distributed servers. This approach ensures real-time alerts and minimizes computational requirements, filling a critical gap in the current research. Unlike previous studies, which typically require substantial infrastructure and processing power, our system is designed to operate effectively in experimental pig facilities with minimal cost and complexity. We need a system that can integrate several types of sensors, video, sound, radar, and proximity, directly on the edge device. Furthermore, by focusing on individual pigs, we can eliminate the confounding factors of social interactions present in group-housed environments, providing more precise and accurate insights into individual animal behavior and welfare. This innovation addresses the limitations of previous work, which has not been applied to single-pig pens or real-time, low-cost monitoring systems. To the best of our knowledge, this has not previously been done in an animal experiment on Göttingen minipigs living in single-pig pens.

The aim of this study is to develop and validate a new pig tracking technology, using video-based data in a machine learning model optimized for low-cost edge device processing to analyze the posture and activity level of experimental pigs living in single-pig pens.

## 2. Materials and Methods

### 2.1. Experimental Site

The model was developed using Göttingen minipigs. These are of a domestic pig breed that is specifically produced for biomedical research due to its unique characteristics with human-like physiology and slow growth, reaching around 40 kg when fully grown. The breed allows for more controlled research and easier handling for long-term-survival pigs. When the research pigs are subjected to operative procedures, they must be kept in a single pen, but near other pigs, with the possibility for snout contact.

The pigs lived in single-pig pens measuring 240 cm × 266 cm in size with six pens in each room at the research facilities, where trained professionals handled the daily care and management of the pigs. The rooms were automatically lit with stable lighting 12 h each day from around 6:30 am to 6:30 pm with pitch-black darkness for the rest of the day.

### 2.2. Component Deployment

A priority in this study was to develop a simple and low-cost system that would be easily applicable in experimental animal research conducted by researchers with limited technical knowledge. This priority was established to secure the highest possible incentive to implement the system in future animal experiments.

**Figure A1:**
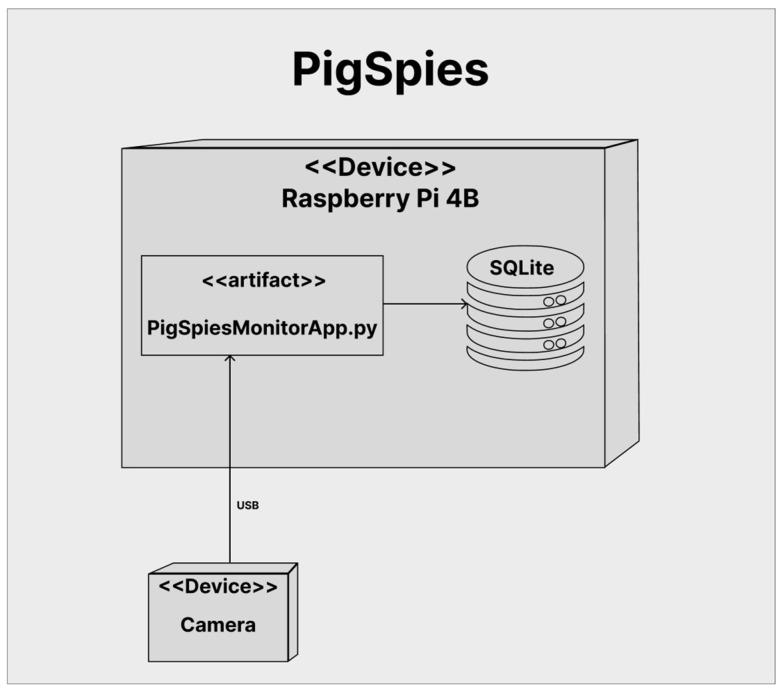
The UML deployment diagram illustrates the Pigspies system. It visualized the system’s hardware and software components.

A camera (USB Camera with Wide-Angle Lens, Marhynchus, Shanghai, China) [15] was mounted using a clamp (Metal Camera Clamp, SMALLRIG Ballhead Clamp, Hong Kong, China) [16] over each pen pointing downwards at an angle of around 10°. The camera was connected to a Raspberry Pi (Raspberry Pi 4B 4 GB, Raspberry Pi Ltd., Cambridge, UK) [17] containing an SD card (SanDisk Extreme 128 GB). The UML deployment diagram is shown in Figure A1 (Appendix A).

### 2.3. Data Collection Platform

A video-based sensor and AI platform named Pigspies was developed to support real-time continuous data capture across six pig pens. A Raspberry Pi Model 4 was selected with a USB video camera, ceiling-mounted with a special metal camera clamp [16]. All cameras were in fixed positions with equal heights and horizontal angles to ensure similar pictures for analysis. A software daemon service for running on Pigspies devices was developed using Python. This included using a video-based object detection YOLO model in combination with context-aware recording software: recordings would start when light was detected and would end when lights were off.

The Pigspies software V1 was developed to run in two modes, data collection for the training of the YOLO model, and real-time for life monitoring. In data collection mode, a folder structure for each day of recording and the wide-angle lens (110°) of the camera were fully utilized to maximize information capture in the pig pen. The video capture framerate was 25 fps. In real time, the framerate was reduced to 1 fps to allow the YOLO model to run in real time.

### 2.4. Image Pre-Processing

During the initial analysis, it was determined that standard image pre-processing techniques were unnecessary for the setting. This conclusion was based on the consistent lighting conditions, fixed camera positioning, and the absence of dynamic shadow artifacts in the recorded footage. As a result, the images did not require additional adjustments or enhancements before analysis.

### 2.5. Activity Monitoring Capability

The activity tracking model was developed using two main algorithms to categorize the state of the pigs. Firstly, the annotation process started using our trained Ultralytics YOLOv8n [18], a model renowned for its rapid processing and high accuracy in object detection. YOLOv8n, known as a ”nano” model, was specifically tailored for edge devices such as the Raspberry Pi 4B [17]. The model identifies whether the pig is lying or standing.

**Figure 1:**
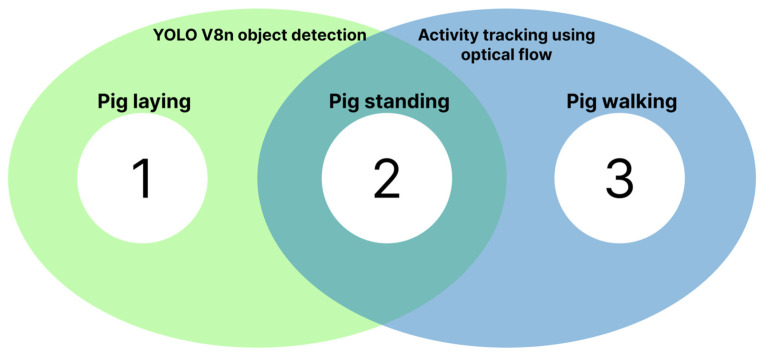
Visual representation of algorithm interactions. The YOLO V8n object detection (green) detects a lying (1) or standing (2) pig. The optical flow analysis (blue) distinguishes if a standing pig is walking (3).

Secondarily, as the next sequential step, if the pig was identified as standing, the Lucas–Kanade sparse optical flow technique distinguished whether the pig was standing still or in movement. Combined, the two algorithms categorized the pig in three different states, lying, standing, or walking, coded as levels 1, 2, and 3, respectively. This is visually represented in Figure 1.

#### 2.5.1. Pig Posture Recognition

**Figure 2:**
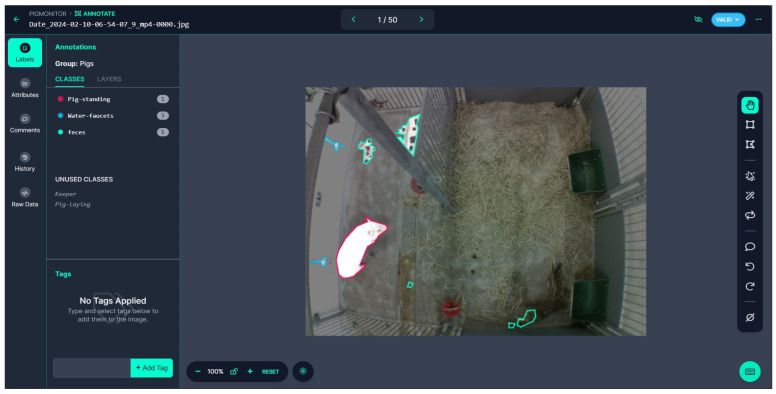
Roboflow annotation tool interface, used for labeling images of a pig enclosure. The left panel lists annotation classes, including “Pig-standing”, “Water-faucets”, and “feces”, along with their respective counts.

**Figure A2:**
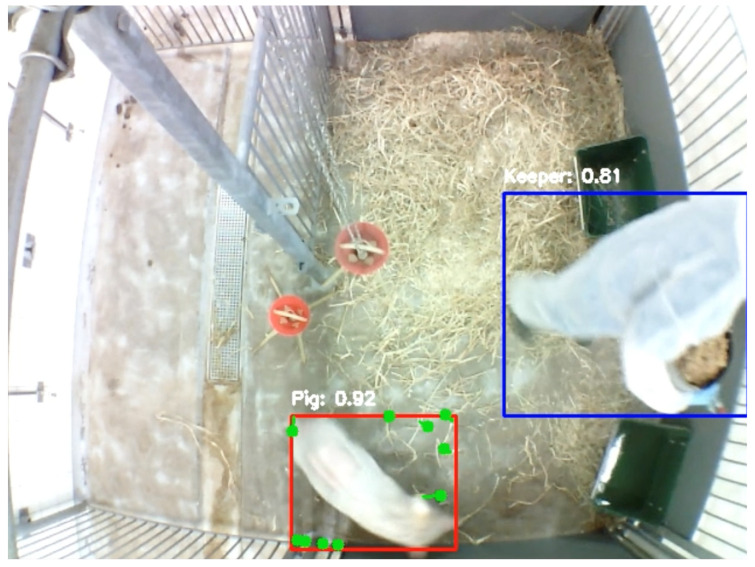
An example of the model’s detection of “Keeper” being a human with the picture frame. This can be used to protect personal data by excluding pictures with a human presence, but also to refine the data from the pig activity, since the pigs will be affected by human presence. For further explanation of colored boxed and green lines and dots, see Figure 7 and Figure 8.

Roboflow [19] software V2024.Q1 was used for the annotation process as seen in Figure 2. Different annotation classes were selected for the model to identify: “Pig-lying”, “Pig-standing”, and “Keeper”. The “Keeper” class is used to identify human presence in the pen. The pig’s activity is influenced by human interactions during feeding, training, examinations, or routine maintenance of the pen. “Keeper” recognition also allows for picture exclusion when humans are present to protect personal data. An example of “Keeper” recognition can be found in Figure A2 (Appendix A).

Following the annotation, the training phase was performed in Google Colab [20] using an Ultralytics Jupyter notebook [21]. Roboflow API was used to fetch the annotated data, and the model was trained on the dataset in the Ultralytics YOLO framework. The result of the extensive training process was a PyTorch model capable of identifying and classifying categories.

#### 2.5.2. Movement Detection

The Lucas–Kanade sparse optical flow algorithm from the OpenCV library [22] was used to track pig movements within a bounding box. This method allows one to focus specifically on the pig’s activities, reducing the influence of other objects or animals in the pen. The bounding boxes isolated the pig from the background and other objects, ensuring that the movement calculations were specific to the pig.

#### 2.5.3. Hyperparameters Used for YOLOv8n Model

The YOLOv8m model used an initial learning rate (lr0) of 0.01 with SGD optimizer, momentum of 0.937, and weight decay of 0.0005. The training ran for 50 epochs with a batch size of 16 and an input image size of 640 × 640. For data augmentation, it employed HSV augmentation (hue: 0.015, saturation: 0.7, value: 0.4), horizontal flipping with 0.5 probability, and mosaic augmentation with 1.0 probability. The model incorporated a learning rate warmup for 3 epochs with a warmup momentum of 0.8 and a warmup bias learning rate of 0.1. The full list of hyperparameters is available in Appendix B.

### 2.6. Framerates

The possibility of analyzing the video in real time was examined. The examination of whether this change in analysis method is feasible was performed using a paired *t*-test in R studio [23]. The test compared the analysis results from two models—the original 25 fps model and a newer model that analyzed 1 fps.

For the 1 fps model, distance thresholds were adjusted to maintain accurate categorization, ensuring that significant movements were still detected and correctly classified. This allowed for precise activity state detection even with the reduced frame rate, ensuring that the model correctly interpreted standing or walking behaviors based on the accumulated distance traveled per second.

## 3. Results

Our major findings consist of (1) model performance, (2) training metrics, (3) validation, (4) longitudinal activity data, and (5) posture recognition.

### 3.1. Model Performance

To illustrate the effectiveness of the training, the following confusion matrix provides a detailed view of the model’s performance.

**Figure 3:**
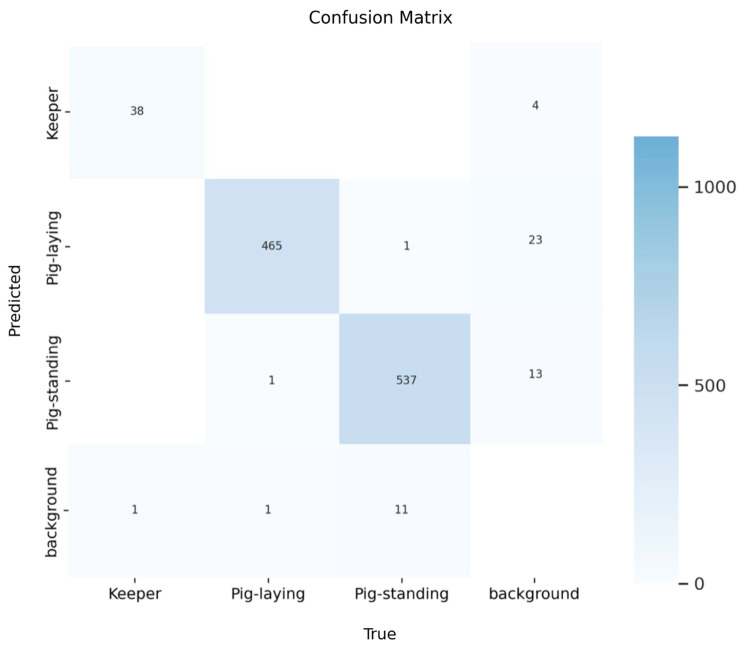
Confusion matrix of the object detection model trained on pigs. The *y*-axis represents the predicted class, while the *x*-axis represents the ground truth observed by the research facilitator.

In Figure 3, the matrix displays a comparison between the true labels (horizontal axis) and the predicted labels (vertical axis). Each cell shows the count of predictions for each specific true-predicted label pair. The diagonal cells represent correct classifications, highlighting the model’s accuracy, while the off-diagonal cells reveal instances of misclassification. The model achieves an overall precision of 93%.

### 3.2. Training Metrics

**Figure 4:**
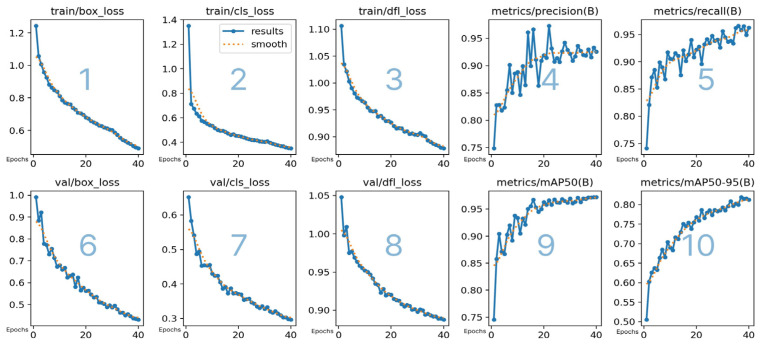
Performance metrics across 40 epochs for the object detection model, illustrating the progression of training and validation losses (box, class, and distribution focal loss) and effectiveness metrics (precision, recall, mean average precision (mAP) at Intersection over Union (IoU) 50%, and mAP from IoU 50% to 95%). These graphs provide insights into the model’s ability to generalize and improve detection accuracy over time, critical for optimizing its deployment in real-world scenarios.

Figure 4 represents training and validation metrics for the Pigspies object detection model. The metrics are described in Table 1.

The graphs in Figure 4 illustrate how the model’s performance changes over the course of 40 epochs, where the epoch is understood as the number of passes the dataset is run in the algorithm.

The training metrics illustrate the model’s performance progression over time in terms of accuracy, precision, and robustness in detecting and classifying objects in images. Declining values in the box, classification, and distribution focal loss metrics suggest that the model is improving its ability to accurately locate and label objects. At the same time, rising precision, recall, and mAP metrics indicate effective reduction of false positives and false negatives, enhancing the model’s reliability.

### 3.3. System Validation

The system categorized pig movements using the numbers 1, 2, and 3, which correspond to lying, standing, and walking, respectively. For validation, these same numerical values were used in a manual posture registration via a dedicated validation script. This approach ensured that human inputs align precisely with the model’s output categories, allowing for a straightforward comparison to verify the model’s categorization accuracy.

#### Validation Results

**Figure 5:**
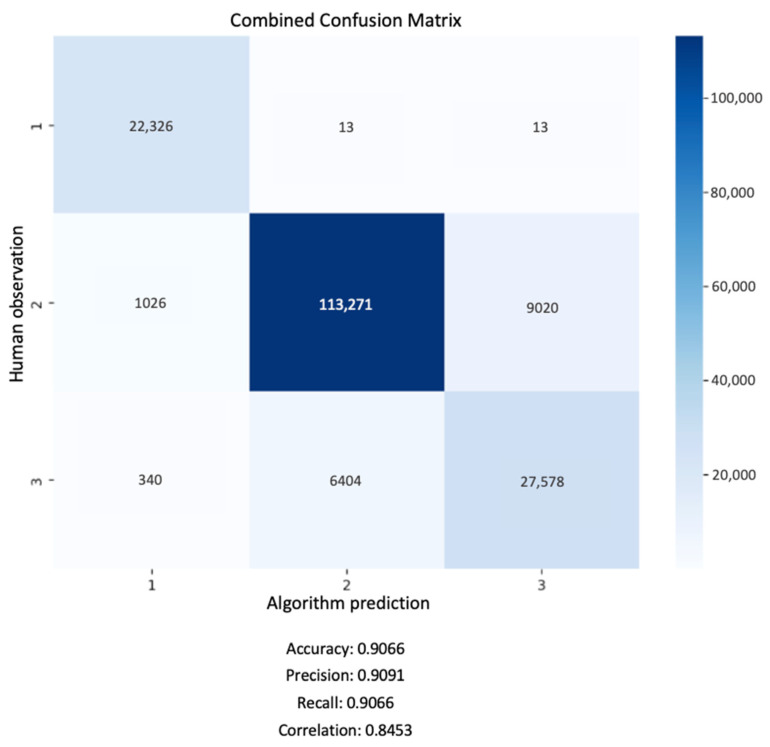
The combined confusion matrix summarizes the results of this validation effort, visually representing the distribution of true and false predictions across three predefined categories.

To assess the accuracy and reliability of the model, a comparison was conducted between the model’s predictions and human observations. From the validation process, the following results were obtained:

The confusion matrix in Figure 5 presents the combined results of the model’s predictions against human observations, considered the ground truth, across all three categories. It details the counts of true positives, false positives, false negatives, and true negatives for each category. As shown in Figure 5, the key performance metrics are an accuracy of 90.66%, precision of 90.91%, recall of 90.66%, and a correlation coefficient of 84.53%.

### 3.4. Activity Levels

**Figure 6:**
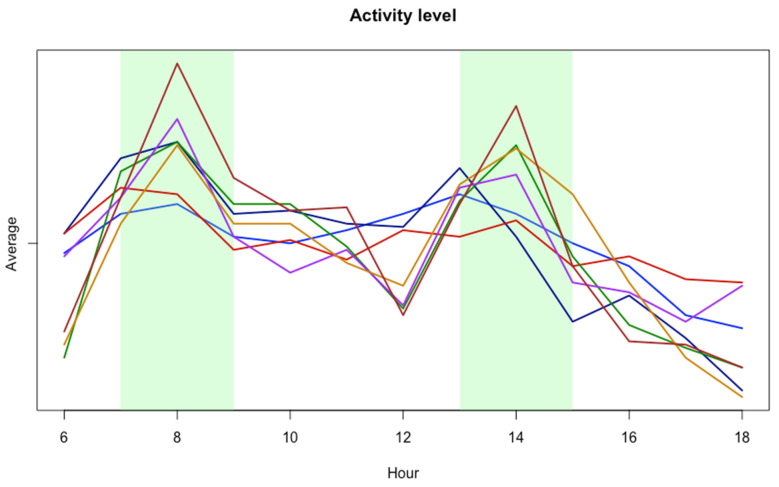
Longitudinal average activity measurements of seven different pigs (colors) for each hour compared to the given pig’s own average activity level from all existing measurements. Green areas represent more active timeslots during the day. Data collection duration ranges from five to eighty days, and all pigs show similar activity patterns.

As shown in Figure 6, all data from seven pigs have been analyzed and the hourly averages of the activity level have been calculated. These are compared to the certain pig’s own average value based on all measured activity independent of day, timepoint, or health status. This is done to surpass inter-pig variation in the baseline values to make comparisons more intuitive.

The figure shows distinct peaks throughout the day, with increases in activity around early morning and mid-afternoon, followed by declines in activity during late morning and evening.

### 3.5. Comparing 25 fps with a 1 fps Algorithm

A comparative analysis of the system’s performance at both 1 fps and 25 fps was conducted using video from 60 h of recording. This involved downscaling the original 25 fps data to 1 fps and comparing the two frame rates through statistical tests.

The paired *t*-test revealed a statistically significant mean difference of 2.1% between the two frame rates (mean difference of 0.021 [CI-95%: 0.0188; 0.0242], *p* < 0.001). Additionally, the binomial test (Table 2) indicated significant differences in the proportions of observed activity levels across frame rates, with *p*-values below 0.001.

### 3.6. Misclassifications

**Figure 7:**
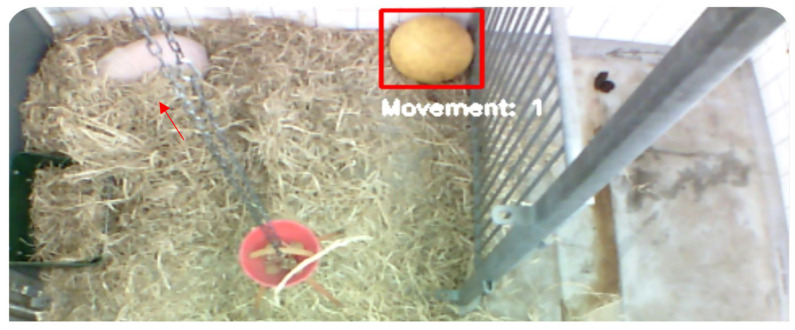
Misclassification example: a ball mistakenly identified as a pig; the actual pig lies under the hay at the top left of the picture (red arrow). The red box indicates that “the pig” is categorized as laying and the Lukas Kanade sparse optical flow is not active.

**Figure 8:**
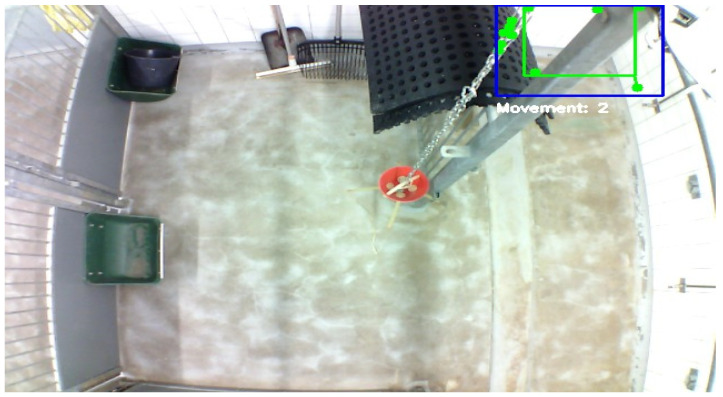
Misclassification example: a section of the wall incorrectly recognized as a pig; no pigs are visible in the scene. The blue box indicates that “the pig” is standing and the Lukas Kanade sparse optical flow is active. The green box identifies the region of interest for further processing using the coordinates of the detected pig’s bounding box. The green vectors and dots represent movement of optical flow features between frames. They show the direction and magnitude of the detected features. The green dots indicate the starting point of the vectors.

The system occasionally misclassifies non-target objects, such as balls (Figure 7) or parts of walls (Figure 8), as pigs. This issue typically arises when no pigs are present or when they are obscured within the video frame.

### 3.7. Posture Recognition

**Figure 9:**
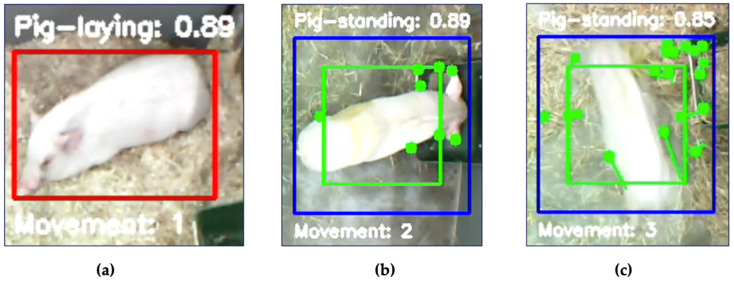
The model successfully distinguished (**a**) class 1, a lying pig, (**b**) from class 2, a standing pig, and (**c**) it registered movement of a non-lying pig, class 3, with sufficient confidence levels above 85%. For explanation of the red, blue, and green boxes, see Figure 7 and Figure 8.

Figure 9 shows examples of the model detecting all three classes with screenshots also including the bounding box around the pig and the confidence level on a scale from 0 to 1 above the bounding box upper border.

## 4. Discussion

### 4.1. Main Outcome

This study developed a monitoring model utilizing a camera setup mounted above each pen. The camera feeds were processed through a Raspberry Pi system that classified pig postures (lying, standing, or walking) with validation from human observation.

The model’s precision in object detection was evaluated over 40 training epochs, using the box, classification, and distribution focal losses metrics. Overall, the training metrics demonstrate the model’s increasing proficiency in object detection, confirming its readiness for practical deployment where consistent and accurate object recognition is essential. The 25 fps model achieved a strong overall performance, with a 93% precision rate, with the main challenges centered on distinguishing the background from objects across all classes. The precision rate of 93% is considered sufficient and corresponds to results from similar articles with comparable but still different methods of pig posture recognition [7,8].

### 4.2. Training and Accuracy

The key performance metrics of the main 25 fps model include an accuracy of 90.66%, a precision of 90.91%, a recall of 90.66%, and a correlation coefficient of 84.53%. While the system’s accuracy of 90.66% is slightly lower compared to similar systems [7], it still demonstrates strong performance and reliability. The accuracy achieved is fully sufficient in clinical/experimental use for observing living species with expected variation in activity patterns also on days in baseline periods. The difference between the observed ground truth and the model’s results could arguably be attributed to the observer not always reacting with the same speed and proficiency as the algorithm, that is, observer bias caused by human inadequacies. The difference in accuracy could also be influenced by the specific dataset used for training, the complexity of the environmental conditions, and the model architecture. Despite the lower accuracy, the system’s results indicate that it is an acceptable tool for monitoring pig activities.

### 4.3. Validation of the Monitoring System for 1 fps

Initially, the system was validated at 25 fps, which provided high detail but did not reflect the typical 1 fps operational rate. Statistical validation using paired *t*-tests and binomial tests revealed only a small mean difference of 2.1% [CI-95%: 0.0188; 0.0242], *p* < 0.001), suggesting acceptable performance variance between frame rates. These findings confirm a statistically significant difference in algorithm performance between 1 fps and 25 fps. However, the practical impact is minimal, with only minor differences in data accuracy and reliability across frame rates, indicating that 1 fps is sufficient for operational use without compromising data integrity.

Future work with testing at 1 fps would offer a more realistic assessment, reinforcing the system’s effectiveness in real-world deployments where accurate behavior monitoring is crucial.

### 4.4. Bounding Boxes

In this model, a bounding box around the pig was used to calculate movement with the Lucas–Kanade sparse optical flow algorithm. Unlike analyzing the entire pen, this method ensures that the activity data specifically reflect the pig’s movements, minimizing the impact of other objects or animals and enhancing data reliability and validity. Validation, however, showed that the system has poor performance, when distinguishing between standing and walking, as seen in Figure 5. This difficulty may result from threshold settings or the need for frame-by-frame human validation to establish a more accurate ground truth. While the bounding box approach allows for precise monitoring, future studies could explore integrating broader pen analysis for a more comprehensive understanding of pig activity and behavior.

### 4.5. Activity Patterns

Preliminary analysis from data collected during the development process could give more insights into the activity patterns of the individual pigs, which can be used in the future to map out how their behavior is influenced by misthriving or discomfort to detect this in future studies. As seen in Figure 6, all seven pigs that were included in long-term data collection showed similar trends, when looking at their hourly activity average during the day. Only complete days of recording were included since the initial days of recording presented us with obstacles. These include Raspberry Pi breakdowns, video storage problems, and non-reliant pig survival. All pigs show increased activity levels during two time periods from 7 a.m. to 9 a.m. and 13 p.m. to 15 p.m. both for working days and weekend days. These trends correspond to several other studies measuring the activity patterns of pigs in different ways [24,25].

It is important to note several differences between this study and the referenced studies that could influence the observed activity patterns. Firstly, the referenced studies use landrace pigs, while this study uses Göttingen minipigs. Additionally, the pigs in the referenced studies were kept together in one pen, while these pigs lived in single-pig pens. The social dynamics of pigs being together versus individually could affect their activity levels. However, legislation and ethics command snout contact between pigs; therefore, no animals lived isolated in rooms. Furthermore, the timing of when the keepers feed the pigs is not necessarily comparable, and in this study, the second feeding is at around 12:15 p.m., which can be seen in the figure as an elevation in the activity level of all pigs at this timepoint. This suggests a benefit of excluding data if humans are present in the future development of the model.

### 4.6. Misclassification of Objects

The misclassifications mentioned in the Results can be attributed to limitations in the training dataset and inherent characteristics of the YOLOv8n model. Specifically, because the dataset lacks a diverse representation of environmental contexts without pigs and includes insufficient examples of potential confounding objects, the model may struggle to accurately differentiate between pigs and objects with similar appearances.

To address the misclassification challenges, several strategies are proposed for future work [21,22]:

1. Enhanced Data Annotation: Expanding the training dataset to include more varied environmental images, particularly those featuring commonly misidentified objects, could enable the model to better distinguish these from the target category.
2. Model Retuning: Adjusting the YOLO model’s parameters or refining it on a more targeted subset of data may improve its discrimination capabilities.
3. Post-processing Algorithms: Integrating additional algorithms to review detected objects based on movement patterns and environmental context could help reduce false positives.
4. Confidence Threshold: Increasing the confidence threshold of the YOLO model may prevent misclassification, such as mistaking a wall for a pig, by requiring a higher certainty for detections to be considered valid.

### 4.7. Challenges with the Raspberry Pi Platform

The Raspberry Pi platform was selected for the monitoring system due to its ease of setup, its cost efficiency, and the straightforward process of duplicating system images across multiple devices. This choice enabled rapid deployment and scalability of the monitoring system. However, challenges with several Raspberry Pi devices shutting down unexpectedly were encountered. To address this problem, several strategies are proposed for future work:

1. Smart Plugs for Easy Management: By using smart plugs, remote management becomes easier, and the Raspberry Pi devices can be restarted remotely or automatically. Keepers and researchers will no longer need to manually reset the gadgets as a result.
2. Cooling Solutions: It is advisable to equip the Raspberry Pi devices with fans to solve any potential overheating concerns that might be causing unplanned shutdowns. Device longevity and stability can be improved with adequate cooling.
3. Regular Restart Scheduling: This will generally help clean up memory leaks and hanging processes. Such restarts should be scheduled frequently, with a frequency of 1 to 24 times a day.

### 4.8. Image Pre-Processing in a Fixed Setup: Justification for Exclusion and Resulting Study Limitations

Image pre-processing is often employed in computer vision tasks to enhance image quality, reduce noise, and prepare data for more accurate analysis. Common pre-processing techniques include resizing, filtering, contrast enhancement, and background subtraction ((Liu et al., 2022) [26], (Maini & Aggarwal 2010) [27], (Gonzalez & Woods, 2018) [28]). While such techniques can be beneficial, their necessity depends on the specific application and the experimental setup.

In this study, image pre-processing was deemed unnecessary due to the controlled nature of the experimental environment and the robustness of the employed algorithms as detailed in the prior sections—YOLO v8n for object detection and Lucas–Kanade sparse optical flow for motion tracking. However, it is a limitation of the study that the results cannot be applied to facilities that rely on more dynamic lighting conditions and with shifting camera angles. The following arguments support this decision:

1. Controlled lighting and camera placement: The experimental setup maintained constant lighting conditions and fixed camera positioning. These factors ensured that the raw images were free from variability caused by external influences, such as shadows, illumination changes, or motion blur. In such stable conditions, the algorithms can directly operate on raw input data without requiring pre-processing steps to mitigate variability. If lighting is not controlled or camera placement is not fixed, this will require additional pre-processing steps. Liu et al. (2022) [26] discuss that fixed or controlled lighting conditions and stable camera positioning reduce the variability in the image data. In such environments, the images captured are less likely to suffer from uneven illumination or perspective distortions, meaning pre-processing for correcting these issues is unnecessary.
2. Robustness of YOLO v8n: YOLO v8n is designed to perform well even with minimal pre-processing. Its built-in capability to handle variations in image quality through convolutional filters ensures that it can accurately detect objects in the controlled environment of this study without pre-processing the input images (Redmon et al., 2016) [29].
3. Lucas–Kanade algorithm assumptions: The Lucas–Kanade sparse optical flow algorithm operates under the assumption of consistent brightness and small motion between frames. These assumptions are satisfied in the fixed experimental setup, arguably making additional pre-processing, such as noise reduction or contrast adjustment, redundant. Moreover, the algorithm’s reliance on tracking feature points rather than pixel-level information further reduces its sensitivity to minor imperfections in raw images (Winkler, 2024) [30]. Again, it is a limitation of the study that this likely cannot be applied to dynamic lightning conditions.
4. Avoiding overhead and potential artifacts: Image pre-processing can introduce artifacts or alter pixel intensity distributions, potentially impacting the performance of both YOLO and optical flow algorithms. By working directly with raw images in a controlled environment, the risk of such artifacts is avoided, ensuring the integrity of the tracking process (Szeliski, 2010) [31].

By leveraging YOLO v8n and the Lucas–Kanade optical flow algorithm with a fixed setup, this study prioritizes computational efficiency and methodological simplicity over the potentially added accuracy, allowing us to run a soft real-time service on the RPI 4B platform. The controlled environment eliminates the variability that would typically necessitate pre-processing, justifying its exclusion, but on the other hand, it arguably reduces its transferability to other research environments with more dynamic light conditions or additional shadowing and other artifacts.

### 4.9. Motivation for Using Lucas–Kanade Sparse Optical Flow Algorithm

The Lucas–Kanade sparse optical flow algorithm was selected for this study due to its alignment with the technical requirements of the experimental setup. This method estimates motion by tracking a sparse set of feature points, which reduces computational demands compared to dense optical flow methods. This is especially relevant on limited computing platforms, like the RPI 4B used in this study. The algorithm assumes small and consistent motion between frames and constant illumination across the scene, making it particularly well suited for setups with stable lighting and fixed camera placement, as is the case in the study facility. Here, lighting is exclusively provided by LED lamps providing the exact same lighting conditions throughout the day. Also, during nighttime, there is complete darkness. Furthermore, the camera is mounted in a fixed position and cannot be moved.

Thus, in this study, lighting levels and camera positions were held constant, ensuring the validity of the brightness constancy assumption inherent to the Lucas–Kanade algorithm. Additionally, the feature-point tracking approach provides robustness against minor occlusions or background inconsistencies, making it a reliable choice for precise movement analysis in controlled environments (Winkler, 2024) [30].

By leveraging the OpenCV library’s implementation of the Lucas–Kanade algorithm, the study benefits from an optimized and widely tested framework, ensuring computational efficiency and reproducibility.

### 4.10. Future Development

#### 4.10.1. Accounting Keeper Presence

To enhance the accuracy of activity monitoring, the system could be evolved to consider the presence of an animal caretaker. Thereby, stratification of data with and without human presence would be possible. Since the object detection model can classify if an object is an animal caretaker, future development should include integrating this capability to adjust activity data analysis. Specifically, the system should differentiate between pig activities when an animal caretaker is present and when pigs are alone. This feature is also important to protect personal data. Adjustments could involve the following:

1. Activity Filtering: Implementing a filtering mechanism to exclude or separately annotate activity data collected while an animal caretaker is detected, ensuring that the pigs’ natural behavior is accurately represented.
2. Behavioral Adjustments: Adjusting behavioral algorithms to account for the presence of keepers, which may temporarily alter pigs’ activity patterns, thus providing a more accurate analysis of their natural behavior.

#### 4.10.2. Twenty-Four-Hour Monitoring

Extending the monitoring to cover a full 24 h period could provide better activity data and a more comprehensive understanding of the pigs’ behavior; however, this is not possible using vision-based picture analysis due to the pitch-black darkness during nighttime in the 12 h controlled environment cycle at the research facilities. Utilizing night vision and other types of heat-sensitive cameras could arguably facilitate effective monitoring during nighttime to capture abnormal nocturnal activity patterns that are currently missed due to the limited monitoring hours. Methods for nocturnal activity tracking using PID sensors are well established and could be implemented in the future [32].

#### 4.10.3. Behavioral Analysis Integration

The developed system could be used to exemplify how observations in animal experiments should be performed in the future, ultimately leading to earlier detection of misthriving, allowing for timely interventions, and reducing pain and suffering in experimental animals. Integrating advanced behavioral analysis algorithms could provide deeper insights into the pigs’ health and well-being. This could include adding more sensors, such as temperature sensors to monitor the pig’s body temperature, water intake sensors to measure the amount of water the pig drinks, and weight sensors to track the pig’s weight. Additionally, incorporating feces assessment to monitor the pig’s digestive health, sensors to measure the amount of urine, and indicators to detect if the pig is sedated could arguably improve the data visualization and overall health monitoring.

#### 4.10.4. Real-Time Deployment

In a real-time deployment with live analysis of the 25 fps video that was retrieved from the camera to the Raspberry Pi, the system faced constraints due to the device’s limited processing power, which could not efficiently handle data analysis at 25 fps. To accommodate this, the analysis was changed to be conducted at 1 fps, which would be a feasible solution to this problem. While this is a reasonable and practical solution to the problem, further optimizations of the algorithms should be considered. Other solutions to this problem could be to use more expensive hardware with stronger processing power. Another solution could be to send the pictures to a server for processing on a more powerful computer.

## 5. Conclusions

A machine-learning-based software system for monitoring and automatically detecting the posture and activity levels of pigs in experimental research facilities was designed, tested, and validated. The software reliably identified key behavioral states (lying, standing, and walking) using a combination of YOLOv8n object detection and the Lucas–Kanade optical flow algorithm.

Validation of the system demonstrated an accuracy of 90.66%, a precision of 90.91%, a recall of 90.66%, and a correlation coefficient of 84.53% when processing video at 25 frames per second. The system’s operational frame rate of 1 fps showed a minor mean difference of 2.1% in performance compared to 25 fps, indicating consistent functionality. This change in operational scheme requires less computer power, leading to increased reliability. While the YOLOv8n model performed well, cases of object misclassification revealed areas for future improvement, including refining object detection algorithms and enhancing activity tracking accuracy when distinguishing standing and walking. Incorporating additional data sources could also enable more comprehensive behavioral insights.

In conclusion, the present study provides valuable contributions to automated pig monitoring using non-invasive machine learning technologies. Further research and engineering are needed to enhance the robustness of the data collection platform and expand its machine-learning-based analytical capabilities to include more fine-grained behavioral assessments.

## Figures and Tables

**Table 1:** Metric descriptions.

Metric	Description
Train/box loss	This graph shows the loss associated with the bounding box predictions during the training phase. The *y*-axis measures the loss magnitude, which ideally decreases over epochs (*x*-axis), indicating the model’s improving accuracy in locating objects.
Train/cls loss	Displays the classification loss during training, measuring how well the model identifies the correct class labels of the objects. The declining curve suggests improving classification accuracy.
Train/dfl loss	Shows the distribution focal loss during the training phase, which focuses on enhancing the model’s accuracy in localizing the exact position of objects. Lower values indicate better localization capabilities.
Metrics/precision (B)	This metric indicates the precision of the model during training, calculated as the ratio of true positive predictions to the total positive predictions made by the model.
Metrics/recall (B)	Reflects the recall rate during training, which measures the model’s ability to identify all actual positives. Higher recall values suggest that the model misses fewer actual positive cases.
Val/box loss	Like the train box loss but for the validation phase. It assesses the model’s ability to generalize bounding box predictions on unseen data.
Val/cls loss	Represents the classification loss during validation. A decreasing trend here is crucial as it signifies effective generalization in classifying new, unseen data.
Val/dfl loss	Mirrors the train distribution focal loss but evaluated on the validation dataset. It checks the model’s performance in object localization under validation conditions.
Metrics/mAP50 (B)	Depicts the mean average precision at 50% Intersection over Union (IoU) threshold during training. This metric assesses the accuracy of the object detector by considering both precision and recall at a moderate IoU threshold.
Metrics/mAP50-95 (B)	Shows the mean average precision across a range of IoU thresholds from 50% to 95% during training. This comprehensive metric evaluates the object detector’s performance across varying levels of detection difficulty, reflecting its effectiveness in various scenarios

**Table 2:** Validation comparison between running at 1 fps and 25 fps.

Variable	Class	Counts	Total	Proportion	*p*
25 fps	1	2,291,225	5,512,255	0.416	<0.001
	2	2,867,129	5,512,255	0.520	<0.001
	3	353,901	5,512,255	0.064	<0.001
1 fps	1	94,247	220,055	0.428	<0.001
	2	113,756	220,055	0.516	<0.001
	3	12,252	220,055	0.056	<0.001

Table 2: Results and proportion comparisons of classes 1, 2, and 3 between the two models showing significant differences in proportions of the classes with *p*-values all under 0.001.

## Data Availability

The data presented in this study are available on request from the corresponding author.

## Appendix B

This section details the hyperparameters used, including an explanation of their role.

Training Hyperparameters

1. task (str): Specifies the task to perform, such as detection, segmentation, classification, or pose estimation.
  o Relevance: Determines the type of model you are training.
2. mode (str): Defines the mode, like training (train), validation (val), or prediction (predict).
  o Relevance: Guides the overall model behavior during the different phases.
3. model (str): Path to the pretrained model.
  o Relevance: Specifies the initial weights or model architecture to use for training.
4. data (str): Path to the dataset configuration file (e.g., COCO or custom dataset).
  o Relevance: Specifies the dataset to use for training or validation.
5. epochs (int): Number of epochs to train the model.
  o Relevance: Defines how many times the model will be trained on the full dataset.
6. patience (int): Number of epochs to wait for improvement before early stopping.
  o Relevance: Helps avoid overfitting and reduces training time if no improvements are seen.
7. batch (int): Batch size, or number of samples per gradient update.
  o Relevance: Controls the amount of data used in each training step and affects training speed and stability.
8. imgsz (int | list): Input image size for training and validation.
  o Relevance: Impacts the performance and accuracy of the model; resizing can reduce computation cost or improve accuracy.
9. save (bool): Whether to save the model checkpoints during training.
  o Relevance: Useful for recovery in case of interruption and for later evaluation or fine-tuning.
10. save_period (int): Frequency (in epochs) for saving model checkpoints.
  o Relevance: Defines how often the model is saved to prevent loss of progress.
11. cache (bool): Whether to use caching for data loading.
  o Relevance: Speeds up training by storing data in RAM or disk.
12. device (str): The device (GPU or CPU) on which to train the model.
  o Relevance: Specifies the hardware used to accelerate training.
13. workers (int): Number of worker threads for data loading.
  o Relevance: Controls the number of parallel processes used for data loading.
14. pretrained (bool): Whether to start with a pretrained model.
  o Relevance: Improves training by leveraging learned features from previous tasks.
15. optimizer (str): The optimizer used for training (e.g., SGD, Adam).
  o Relevance: Controls how the model’s weights are updated during training.
16. verbose (bool): Whether to output detailed logs during training.
  o Relevance: Provides more insights into the training process.
17. seed (int): Random seed for reproducibility.
  o Relevance: Ensures consistent results across runs.
18. deterministic (bool): If set to True, forces deterministic operations.
  o Relevance: Ensures that results are consistent across different hardware configurations.
19. single_cls (bool): Whether to treat all classes as a single class.
  o Relevance: Useful for training on single-class datasets.
20. rect (bool): Whether to use rectangular images during training.
  o Relevance: Optimizes the training process for rectangular images, reducing padding.
21. cos_lr (bool): Whether to use a cosine learning rate scheduler.
  o Relevance: Helps to gradually decrease the learning rate, potentially improving convergence.
22. resume (bool): Whether to resume training from the last checkpoint.
  o Relevance: Allows the continuation of training from a previous state.
23. amp (bool): Whether to use automatic mixed precision for training.
  o Relevance: Speeds up training by using reduced-precision floating-point operations.
24. fraction (float): Fraction of the dataset to use for training.
  o Relevance: Useful for debugging or training on a subset of the data.
25. multi_scale (bool): Whether to train the model on images of varying scales.
  o Relevance: Makes the model more robust to different object sizes.

Hyperparameters for Optimizer and Loss

1. lr0 (float): Initial learning rate.
  o Relevance: Controls how fast the model learns. A higher value may lead to faster convergence but instability.
2. lrf (float): Final learning rate as a fraction of lr0.
  o Relevance: Gradually decreases the learning rate for smoother convergence.
3. momentum (float): Momentum factor for optimizers.
  o Relevance: Helps accelerate gradients vectors in the right directions, improving convergence.
4. weight_decay (float): Weight decay (L2 regularization) to prevent overfitting.
  o Relevance: Regularizes the model to avoid large weights and reduce overfitting.
5. warmup_epochs (float): Number of epochs for warmup.
  o Relevance: Gradually increases the learning rate at the start of training to avoid instability.
6. warmup_momentum (float): Initial momentum during warmup.
  o Relevance: Helps smooth out early training by avoiding sudden jumps in momentum.
7. box, cls, dfl, pose, kobj (float): Loss gains for different components of the model.
  o Relevance: Balances the contribution of box loss, class loss, keypoint loss, etc., during training.
8. label_smoothing (float): Degree of label smoothing.
  o Relevance: Helps prevent overfitting by softening the target labels.

Data Augmentation

1. hsv_h, hsv_s, hsv_v (float): Augmentation parameters for image Hue, Saturation, and Value.
  o Relevance: Introduces color variation into the training data, improving robustness.
2. degrees (float): Range of random rotations.
  o Relevance: Adds rotation variation to the dataset.
3. translate (float): Translation range for shifting images.
  o Relevance: Simulates different object positions within the image.
4. scale (float): Scaling factor for resizing images.
  o Relevance: Augments the dataset with different object sizes.
5. shear (float): Shearing range for perspective distortions.
  o Relevance: Adds variety to image distortions, helping with model robustness.
6. flipud, fliplr (float): Probability for flipping images vertically or horizontally.
  o Relevance: Increases dataset diversity by simulating mirror images.
7. mosaic (float): Probability of using mosaic augmentation.
  o Relevance: Combines multiple images into one for increased dataset diversity.
8. mixup (float): Probability of mixing images for augmentation.
  o Relevance: Generates synthetic data to improve robustness.

Other Settings

1. tracker (str): Tracker type
  o Relevance: Defines the tracker to use during object tracking in videos.

These hyperparameters are critical for controlling various aspects of training, such as learning rates, regularization, data augmentation, and model behavior, ensuring the effective training and evaluation of the YOLO model.
